# Endoscopic surgery versus intensity-modulated radiotherapy in locally advanced recurrent nasopharyngeal carcinoma: a multicenter, case-matched comparison

**DOI:** 10.1186/s40463-023-00656-3

**Published:** 2023-11-06

**Authors:** Yibin Liu, Nan Huang, Junxiao Gao, Bin He, Hongming Huang, Liangcai Wan, Qinming Cai, Zhenchao Zhu, Suizi Zhou, Jing Wang, Xiaohui Wang, Qianhui Qiu, Fei Han

**Affiliations:** 1 Department of Otolaryngology and Head and Neck Surgery, Guangdong Provincial People’s Hospital (Guangdong Academy of Medical Sciences), Southern Medical University, Guangzhou, China; 2 Department of Radiation Oncology, State Key Laboratory of Oncology in South China, Guangdong Key Laboratory of Nasopharyngeal Carcinoma Diagnosis and Therapy, Guangdong Provincial Clinical Research Center for Cancer, Sun Yat-sen University Cancer Center, Guangzhou, China; 3 Department of Otolaryngology and Head and Neck Surgery, Zhujiang Hospital, Southern Medical University, Guangzhou, China; 4 Department of Otolaryngology and Head and Neck Surgery, Wuzhou Red Cross Hospital, Wuzhou, China

**Keywords:** Nasopharyngeal carcinoma, Recurrent, Surgery, Radiotherapy

## Abstract

**Background:**

The management of locally advanced recurrent nasopharyngeal carcinoma (rNPC) is challenging. The objective of our study was to compare salvage endoscopic nasopharyngectomy (ENPG) with intensity-modulated radiotherapy (IMRT) in clinical outcomes and complications of locally advanced rNPC.

**Methods:**

Patients with histologically confirmed rNPC in rT_3-4_N_0-3_M_0_ stages were retrospectively enrolled between January 2013 and December 2019 in this multicenter, case-matched study. The baseline clinicopathological characteristics of patients were balanced by propensity score matching between the ENPG and IMRT groups. ENPG was performed in patients with easily or potentially resectable tumors. The oncological outcomes as well as treatment-related complications were compared between two groups.

**Results:**

A total of 176 patients were enrolled and 106 patients were matched. The ENPG group (n = 53) and the IMRT group (n = 53) showed comparable outcomes in the 3-year overall survival rate (68.4% vs. 65.4%, *P* = 0.401), cancer-specific survival rate (80.9% vs. 74.4%, *P* = 0.076), locoregional failure-free survival rate (36.6% vs. 45.3%, *P* = 0.076), and progression-free survival rate (27.5% vs. 32.3%, *P* = 0.216). The incidence of severe treatment-related complications of patients in the ENPG group was lower than that in the IMRT group (37.7% vs. 67.9%, *P* = 0.002). The most common complications were post perioperative hemorrhage (13.2%) in ENPG group and temporal lobe necrosis (47.2%) in IMRT group, respectively.

**Conclusion:**

Salvage ENPG exhibits comparable efficacy but less toxicities than IMRT in carefully screened patients with locally advanced rNPC, which may be a new choice of local treatment.

**Graphical abstract:**

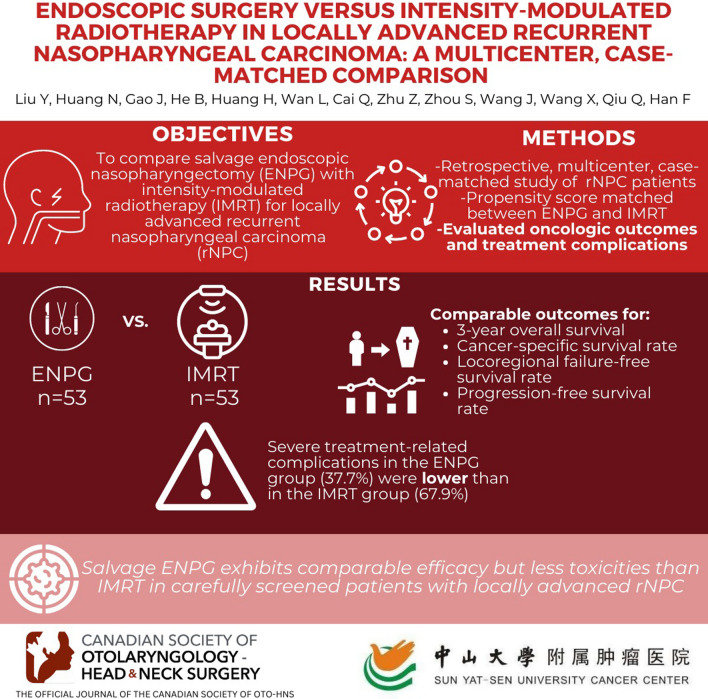

**Supplementary Information:**

The online version contains supplementary material available at 10.1186/s40463-023-00656-3.

## Background

Nasopharyngeal carcinoma (NPC) is a malignancy originating from the nasopharyngeal mucosal lining. NPC has a highly unbalanced geological distribution worldwide; cases are mainly concentrated in southeast Asia, especially in south China [1]. According to the report of GLOBCAN in 2020, the numbers of newly diagnosed cases and deaths are 133,354 and 80,008, respectively [2]. Radiotherapy (RT) and chemoradiotherapy are the mainstay treatments for primary NPC due to its sensitivity to these modalities. Since the popularization of intensity-modulated radiotherapy (IMRT), the clinical outcomes of patients with NPC have greatly improved. The 5-year overall survival (OS) rate of the whole population is approximately 80%, and that of patients in early stages has surpassed 90% [3]. However, approximately 10 ~ 20% of patients will experience locoregional recurrence after undergoing standard treatment [3–5].

The management of recurrent NPC (rNPC) is challenging. The recommended therapeutic modalities for rNPC include reirradiation, surgery, chemotherapy, targeted therapy and immunotherapy, and the former two are potentially curative therapies. Reirradiation with IMRT is superior to conventional two-dimensional RT and three-dimensional conformal RT (3D-CRT) in dose conformity and target homogeneity as well as sparing organs at risk (OARs). In addition, IMRT can be used to treat more extensive lesions than brachytherapy and stereotactic radiosurgery. Therefore, reirradiation with IMRT is applied to rNPC in all stages and has greatly prolonged patient survival, with a 5-year overall survival (OS) rate of 36.6% to 67.2% [6–8]. However, cumulative doses delivered by two courses of RT add the possibility of radiation-induced toxicities of normal tissues.

To address this issue, the role of salvage surgery in rNPC has become increasingly prominent, as it can resect radioresistant tumor tissues and avoid the accumulation of radiation-related toxicities. Although conventional nasopharyngectomy with an extranasal approach has achieved similar clinical outcomes compared with reirradiation, with a 5-year OS rate of 30% to 60%, great damage to normal structures and severe complications such as maxillary necrosis and palatal fistula preclude further clinical practice [9, 10]. Endoscopic nasopharyngectomy (ENPG) possesses the advantages of less structural damage, no facial incision and rapid postoperative recovery by using the natural cavity as the operation path and can obtain almost the same resection extent as open surgery [11]. A growing amount of evidence has proven its efficacy in treating rNPC. Recently, a multicenter, randomized, controlled phase III clinical trial reported that for patients with resectable local rNPC (rT_1-2_, selective rT_3_), ENPG exhibited a significant survival benefit over IMRT (3-year OS: 85.5% vs. 68.0%, *P* = 0.0015) [12]. Herein, it is recommended that ENPG should be given priority for resectable lesions [13]. However, the majority of recurrent patients are diagnosed with locally advanced disease [14, 15]. For most patients in the rT_3_ stage and all patients in the rT_4_ stage, a tumor with a large volume often invades the skull base or intracranial structures and is adjacent to or wraps around the internal carotid artery (ICA), making en bloc resection difficult and risky. Thus, the role of ENPG in locally advanced rNPC needs to be further explored.

With the better acknowledgment of endoscopic skull base anatomy and the wide application of advanced medical instruments such as navigation and skull base drilling, the ENPG technique is becoming increasingly mature. Experienced surgeons can remove lesions involving the parapharyngeal space, intracranial cavernous sinus and ICA, which greatly improves the resection rate of locally advanced rNPC. Moreover, with the addition of postoperative RT or adjuvant chemotherapy, immunotherapy and targeted therapy, patients can achieve long-term survival and a better quality of life [16–18]. Nevertheless, these current studies have the limitations of small sample sizes or only being single-arm studies. ENPG in locally advanced rNPC lacks high-level evidence, especially its efficacy and safety profile compared with currently acknowledged IMRT, which needs further research to confirm. Herein, we conducted a multicenter, case-matched study to compare salvage ENPG with IMRT in clinical outcomes and complications of patients in rT_3-4_ stages.

## Methods

### Patients

In this study, we retrospectively included rNPC patients diagnosed between January 2013 and December 2019 from four hospitals in South China (Additional file 1: Table 1). The demographics, clinical characteristics and treatment data of the patients were retrieved through electronic medical record systems. The patients were re-staged according to the 8th edition of TNM staging criteria for NPC established by the American Joint Committee on Cancer. All patients previously provided written informed consent before salvage treatment. The work was carried out in accordance with the Declaration of Helsinki. Ethics approval for this retrospective study was obtained from the four hospitals.

**Table 1:** Baseline demographics and clinical characteristics of the patients after matching

Characteristics	Overall	ENPG	IMRT	*P*-value
	NO. (%)	NO. (%)	NO. (%)	
No. of patients	106	53	53	
Sex				0.791
Male	89 (84.0)	45 (84.9)	44 (83.0)	
Female	17 (16.0)	8 (15.1)	9 (17.0)	
Median age at recurrence, years (IQR)	48 (40–56)	49 (39–55)	47 (40–57)	0.992
Median Recurrent-free interval, months (IQR)	36 (20–73)	32 (13–79)	40 (23–61)	0.355
Histology				0.780
WHO type II + III	91 (85.8)	45 (84.9)	46 (86.8)	
WHO type I + others	15 (14.2)	8 (15.1)	7 (13.2)	
Recurrent T classification				1.000
rT3	56 (52.8)	28 (52.8)	28 (52.8)	
rT4	50 (47.2)	25 (47.2)	25 (47.2)	
Recurrent N classification				0.172
rN0	75 (70.8)	42 (79.2)	33 (62.2)	
rN1	21 (19.8)	3 (5.7)	18 (34.0)	
rN2	8 (7.5)	7 (13.2)	1 (1.9)	
rN3	2 (1.9)	1 (1.9)	1 (1.9)	
Systemic treatment				0.807
Yes	85 (80.2)	43 (81.1)	42 (79.2)	
No	21 (19.8)	10 (18.9)	11 (20.8)	

*IQR* Inter quartile range; *ENPG* endoscopic nasopharyngectomy; *IMRT* intensity-modulated radiotherapy

The inclusion criteria were as follows: primary tumor undergoing definitive RT; clinical stage of rT_3-4_N_0-3_M_0_ at recurrence; age at recurrence between 18 and 70 years; a Karnofsky Performance Status score of at least 70; sufficient organ function; at least a 6-month recurrence-free interval (RFI, defined as the interval between the completion date of initial course of RT to recurrence); ENPG group undergoing salvage ENPG with or without systemic therapy and IMRT group undergoing salvage IMRT with or without systemic therapy within 6 months after recurrence; and permission to receive cross-group therapy after salvage treatment failure.

The exclusion criteria were as follows: the presence of nasopharyngeal mucosal necrosis in the IMRT group; pathological unproven NPC; distant metastasis at recurrence; previous history of other malignancies; no follow-up records after salvage treatment. Meanwhile, recurrent tumors were carefully evaluated by experienced surgeons and radiation oncologists. Patients with unresectable tumors including (1) intracranial invasion of the functional brain area, (2) invasion of the posterior of the cervical vertebra, (3) ICA invasion without embolization or stent intervention and (4) irreparable huge defects after resection, were excluded from the ENPG group.

### Treatment

All patients in the ENPG group underwent endoscopic transpterygoid nasopharyngectomy based on Stamm’s surgical approach [19]. If the tumor involved the cavernous sinus (CS) or ICA, the detailed resection procedures were as follows. The root of the pterygoid process and anterior and lateral walls of the sphenoid sinus were removed with a high-speed drill to further expose the anterior, lateral and inferior walls of the CS and ICA clival segments. The ICA was dissected from the upper to the horizontal section and from the lower to the foramen lacerum; the petrous segment was also dissected if necessary. After separation from top to bottom along the lateral side of the ICA clivus segment, a small incision was made at the anterior wall of the CS along the medial edge of the ICA CS segment and then the CS was directly entered to separate and expand the incision outward. If the operative field was poorly exposed, a second incision was made perpendicular to the first incision to the lateral edge of the ICA CS segment [20]. Since most of the blood sinuses were occupied or squeezed by the tumor after the invasion of the CS, generally, the bleeding was not severe and could be controlled by temporary tamponade with absorbable hemostat. Subsequently, the tumor in the CS was removed mainly by scraping with a curette. If the tumor invaded the ICA, segmental artery resection was performed on the premise of embolizing the ICA beforehand or protecting the ICA with a stent during the operation. Then, the exposed area of the ICA and other important parts were repaired with a nasal septum pedicled mucosal flap, turbinate mucosal flap, temporal muscle flap or artificial skin.

We also performed radical neck dissection for the patients with concurrent neck recurrence. During the operation, the surgeon should try to obtain negative surgical margins and negative postoperative images as much as possible; if not, then chemotherapy, targeted therapy or immunotherapy should be recommended after the operation. The patients with tumor progression or recurrence who were suitable for surgery were encouraged to undergo endoscopic surgery again; those who were not suitable for surgery but suitable for radiotherapy underwent IMRT; and the remaining patients received chemotherapy, targeted therapy, immunotherapy, or best supportive therapy.

The procedure of reirradiation with IMRT was in accordance with previous reports [12, 21–23]. The patients were immobilized from the head, neck and shoulders with a thermoplastic mask while they were in the supine position. Then, CT images extending from the vertex to 2 cm below the sternoclavicular joint with a slice thickness of 3 mm were obtained for delineation of target volumes and OARs. According to the International Commission on Radiation Units and Measurements reports 50 and 62, the recurrent gross tumor volume (rGTV) in the nasopharynx (rGTVnx) and metastatic lymph nodes (rGTVnd) were determined by MRI or CT or 18F-fluorodeoxyglucose PET-CT and physical examinations; the recurrent clinical target volume (rCTV) was contoured as the rGTV plus a 5–10 mm margin or a smaller margin (< 3 mm) if the tumor was adjacent to critical OARs, e.g., the brainstem and spinal cord. We prescribed a dose of 60 ~ 66 Gy to the rGTVnx, and 54 Gy to the rCTV, all in 27 ~ 33 fractions. Dose constraints of the OARs were determined by RFI and the standard threshold doses of normal structures. All patients completed the reirradiation as planned, and chemotherapy, targeted therapy, immunotherapy and ENPG were used as adjuvant or subsequent-line treatments at the physician’s discretion.

### Follow-up

After completing treatment, the patients in the two groups were followed up at least every 3 months during the first 2 years and every 6 months thereafter according to the same standard protocol. Particularly, the patients in the ENPG group were recalled weekly to clean the operative cavity during the first month after surgery. The patients were evaluated by regular nasopharyngoscopy, nasopharyngeal plus cervical MRI, thoracic plus abdominal CT and bone scan. We recorded survival status, causes of death, locoregional recurrence, adjuvant or subsequent-line treatment and severe adverse events. Late radiation-induced toxicities were graded based on the Radiation Therapy Oncology Group and European Organization for Research and Treatment of Cancer late radiation morbidity scoring scheme, and ENPG-related complications were graded according to the National Cancer Institute Common Terminology Criteria for Adverse Events version 5.0.

### Statistical analysis

To balance the covariates irrelevant to local salvage treatment, we performed the propensity score matching (PSM) method at a 1: 1 ratio to adjust for sex, age, pathology, rT and rN stage, with or without systemic treatment and RFI between the ENPG and IMRT groups. Locoregional failure-free survival (LRFFS) was calculated from the date of radiological recurrence to the date of locoregional failure. Progression-free survival (PFS) was defined as the time from recurrence to disease progression or death from any cause. OS was defined as the time from recurrence to death from any cause. Cancer-specific survival (CSS) was defined as the time from recurrence to death from NPC. We used the Kaplan–Meier method to analyze survival data and compared the survival difference by the log-rank test. Univariate and multivariate analyses were performed by using the Cox proportional hazards model. For continuous variables, an independent t test was conducted for normally distributed data; otherwise, the Mann–Whitney U test was performed. Categorical variables were analyzed using the chi-square test or Fisher’s exact test. Statistical analyses were performed using SPSS software (version 26.0, SPSS Inc., Chicago, IL), and a two-sided *P* value < 0 0.05 was considered significant.

## Results

### Clinicopathological characteristics of the patients

**Fig. 1:**
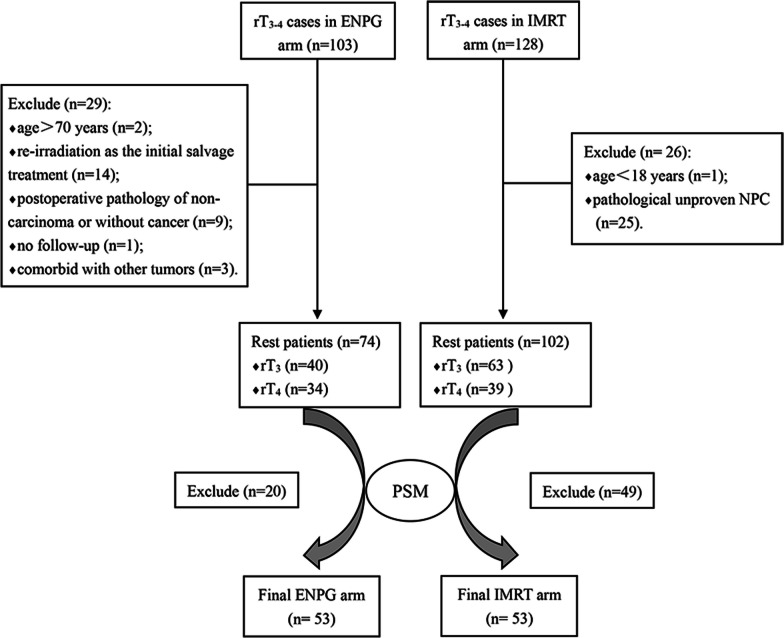
CONSORT flow diagram. *PSM* Propensity score matching. *rNPC* recurrent nasopharyngeal carcinoma

A total of 231 patients with locally advanced rNPC were evaluated for eligibility, including 103 patients in ENPG group and 128 patients in the IMRT group. According to the inclusion and exclusion criteria, we finally enrolled 74 and 102 patients in the ENPG and IMRT groups, respectively (Fig. 1). The baseline demographics and clinical characteristics of the two groups are listed in Additional file 2: Table 2; the two groups differed in pathology, rN stage and with or without systemic treatment. After adjustment by the PSM method, the 7 factors were well balanced between the ENPG (n = 53) and IMRT (n = 53) groups (Table 1). Theoretically, reirradiation with IMRT is applicable for almost all rT_3-4_ rNPC patients, while the extent of the involved structures will largely determine the difficulty of resection. Hence, relying on high-resolution contrast MRI and current evidence as well as our experience, we carefully identified the anatomical structures the recurrent tumor had invaded and classified them into easily resectable lesions and potentially resectable lesions. The involved structures of every patient in the matched cohort (n = 106) are recorded in Table 2.

**Table 2:** The invaded structures classified by the difficulty of surgical resection

Category	Invaded structures	ENPG	IMRT	*P* value
Easily resectable tumors	Confined to base wall of the sphenoid sinus	19	29	0.612
	Superficial clivus	21	30	
	Ethmoid sinus	11	7	
	Maxillary sinus	3	4	
	Pterygoid structures	38	46	
Potentially resectable tumors	Beyond base wall of the sphenoid sinus	18	21	0.431
	Inner table of the clivus	17	14	
	Petrous apex	30	41	
	ICA	14	23	
	Meninges	6	8	
	Cavernous sinus	17	24	
	Non-functional brain area	3	1	
	Orbit	7	2	
	Optic nerve	3	2	

*Pterygoid structures* including medial/lateral plate of pterygoid process, root of pterygoid process, maxillary fissure of pterygoid process, and pterygopalatine fossa; *ICA* Internal Carotid Artery

In the matched ENPG group (n = 53), 5 patients with radiological neck recurrence underwent radical neck dissection; pathological lymph node metastasis was confirmed in 4 patients. There were 38 (71.7%) patients with positive surgical margins and/or positive postoperative images; only 27 patients received adjuvant chemotherapy, targeted therapy or immunotherapy. For the 30 patients with persistent or recurrent locoregional disease after salvage ENPG, the subsequent treatment is summarized in Table 3. Skull base reconstruction was performed in 35 patients, including pedicled nasal septum mucosal flap in 17 patients (32.1%), pedicled inferior turbinate mucosal flap in 1 patient (1.9%), temporal muscle flap in 1 patient (1.9%), free turbinate mucosa in 12 patients (22.6%), thigh myofascial in 1 patient (1.9%) and artificial skin in 3 patients (5.7%); 18 patients (34.0%) remained unrepaired. In the matched IMRT group (n = 53), 22 patients experienced locoregional residue or recurrence after salvage IMRT, and their subsequent treatment is shown in Table 3.

**Table 3:** The subsequent-line treatment of patients with persistent or recurrent local–regional disease

Treatment	ENPG (n = 53)	IMRT (n = 53)
ENPG alone	9	3
IMRT alone	1	1
ENPG + systemic treatment	9	4
ENPG + IMRT + systemic treatment	2	0
ENPG + IMRT	0	0
Systemic treatment	4	11
Supportive treatment	5	3
Total	30	22

*ENPG* endoscopic nasopharyngectomy; *IMRT* intensity-modulated radiotherapy; systemic treatment including chemotherapy or targeted therapy or immunotherapy

### Survival outcomes

The last follow-up was on 30 September 2021, and the median follow-up duration was 30.4 (interquantile range, IQR 18.6–42.1) months. Five and 14 patients in the ENPG and IMRT groups were lost to follow-up, respectively. Twenty-two deaths occurred in the ENPG group (n = 53) and 11 patients died of tumor progression, 5 patients died of nasopharyngeal hemorrhage, 1 patient died of acute respiratory failure, 1 patient died of severe pneumonia, 1 patient died of neurogenic shock, 1 patient died of accidental choking and 2 patients died of unknown causes. Among the 22 dead patients in the ENPG group, 18 (81.8%) had positive surgical margins and/or positive postoperative images, and the operative area was not reconstructed in 10 patients (45.5%). Nineteen of the dead patients received adjuvant therapy after ENPG, including radiotherapy alone (n = 12), radiotherapy plus chemotherapy or immunotherapy (n = 6) and chemotherapy plus immunotherapy (n = 1). Twenty-three patients in the IMRT group (n = 53) died and 6 patients died of residual disease, 4 patients died of local recurrence, 2 patients died of distant metastasis, 6 patients died of radiation-related toxicities, 1 patient died of intracranial infection and 4 patients died of unknown causes.

**Fig. 2:**
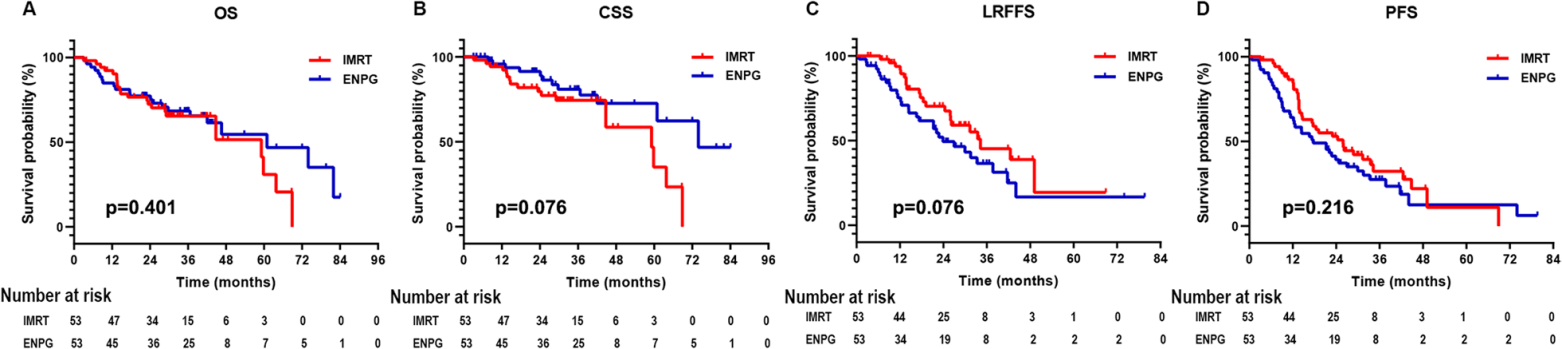
The survival outcomes of patients in the ENPG and IMRT groups. *ENPG* endoscopic nasopharyngectomy; *IMRT* intensity-modulated radiotherapy

The survival rates of the patients in the matched cohort (n = 106) are summarized in Table 4, and survival curves are displayed in Fig. 2. The 3-year OS, CSS, LRFFS and PFS rate of patients in ENPG group was 68.4%, 80.9%, 36.6% and 27.5%, respectively, compared with 65.5%, 74.4%, 45.3% and 32.3% in the IMRT group; no statistically significant difference was found between the ENPG group and the IMRT group in OS rate (*P* = 0.401), CSS rate (*P* = 0.076), LRFFS rate (*P* = 0.076) and PFS rate (*P* = 0.216).

**Table 4:** The survival rates of patients in the matched cohort

Survival rates	ENPG (n = 53)	IMRT (n = 53)	*P*-value
OS			0.401
Deaths	22 (41.5%)	23 (56.6%)	
1-year	84.9%	92.3%	
2-year	75.2%	72.5%	
3-year	68.4%	65.4%	
CSS			0.076
Cancer-specific death	12 (22.6%)	18 (34.0%)	
1-year	95.8%	94.3%	
2-year	88.9%	79.6%	
3-year	80.9%	74.4%	
LRFFS			0.076
Locoregional failure	30 (56.6%)	22 (41.5%)	
1-year	75.4%	91.7%	
2-year	49.5%	67.6%	
3-year	36.6%	45.3%	
PFS			0.216
Disease progression or death	41 (77.4%)	36 (67.9%)	
1-year	64.2%	84.6%	
2-year	39.3%	50.9%	
3-year	27.5%	32.3%	

*ENPG* endoscopic nasopharyngectomy; *IMRT* intensity-modulated radiotherapy; *OS* overall survival; *CSS* cancer-specific survival; *LRFFS* local–regional failure free survival; *PFS* progression free survival

### Treatment-related complications

Treatment-related complications were assessed in the matched cohort (n = 106, Table 5). Generally, twenty (37.7%) and 36 (67.9%) patients in the ENPG and IMRT groups experienced 1 or more types of grade 3 or worse adverse events. Temporal lobe necrosis and mucosal necrosis were the most common late radiation-induced complications, and their incidences were significantly higher in the IMRT group than in the ENPG group (47.2% vs. 15.1%, *P* < 0.001; 26.4% vs. 3.8%, *P* = 0.001). The adverse events of grade 3 or worse that were specific to surgery included 3 cases of perioperative hemorrhage and 1 case of wound infection. No perioperative deaths occurred. Seven patients had post perioperative hemorrhage and 4 patients died. The median amount of intraoperative bleeding was 300 (IQR 135–325) ml. Four patients with a negative balloon occlusion test (BOT) underwent ICA embolization, and 1 experienced large- area cerebral infarction.

**Table 5:** Severe treatment-related complications of patients in the matched cohort

Severe treatment-related complications	ENPG (n = 53)	IMRT (n = 53)	*P*-value
Adverse events specific to RT			
Hearing loss (grade ≥ 3)	2 (3.8%)	4 (7.5%)	0.678
Mucosal necrosis	2 (3.8%)	14 (26.4%)	0.001
Hemorrhage	1 (1.9%)	4 (7.5%)	0.363
Trismus (≤ 1 cm)	1 (1.9%)	4 (7.5%)	0.363
Temporal lobe necrosis	8 (15.1%)	25 (47.2%)	< 0.001
Xerostomia (grade ≥ 3)	0	0	
Adverse events specific to surgery			
Perioperative hemorrhage	3 (5.7%)	0	
Wound infection	1 (1.9%)	0	
Turbinate synechiae	0	0	
Post perioperative hemorrhage	7 (13.2%)	0	
Cranial nerve palsy	1 (1.9%)	5(9.4%)	0.205
Overall	20 (37.7%)	36 (67.9%)	0.002

*ENPG* endoscopic nasopharyngectomy; *IMRT* intensity-modulated radiotherapy; *RT* radiotherapy

### Prognostic factors

A univariate analysis was performed to explore the potential prognosticators of OS in the matched cohort. As shown in Table 6, only younger age and pathological subtype of non-keratinizing carcinoma were significantly correlated with favorable OS (both *P* < 0.05). Then, age and pathology were introduced into the multivariate Cox regression model and age was proven to be an independent prognostic factor for OS (*P* = 0.006).

**Table 6:** Univariate and multivariate analyses of potential prognostic factors on OS in the matched cohort

Variables	Univariate	Multivariate		
	HR (95%CI)	*P*-value	HR (95%CI)	*P*-value
Treatment				
IMRT	Reference			
ENPG	0.772 (0.421–1.414)	0.402		
Age at recurrence	1.049 (1.017–1.083)	0.003	1.046 (1.013–1.081)	0.006
Pathology				
Non-keratinizing carcinoma	Reference			
Others	2.296 (1.091–4.834)	0.029	1.955 (0.924–4.137)	0.080
Sex				
Female	Reference			
Male	0.887 (0.393–2.002)	0.774		
Recurrent-free interval	1.001 (0.996–1.006)	0.711		
Recurrent T classification				
rT_3_	Reference			
rT_4_	1.525 (0.844–2.757)	0.162		
Recurrent N classification				
rN_0_	Reference			
rN_1-3_	0.880 (0.441–1.756)	0.716		
Systemic treatment				
No	Reference			
Yes	1.071 (0.475–2.411)	0.869		

*OS* overall survival; *ENPG* endoscopic nasopharyngectomy; *IMRT* intensity-modulated radiotherapy

## Discussion

Reirradiation is currently regarded as the main local treatment for locally advanced rNPC. Our study showed that the prognosis of patients in rT_3-4_ stages was greatly improved in the IMRT era owing to the improvement of radiotherapy techniques and the modified total dose and fraction size compared with previous studies [24, 25]. For instance, 54.7% (29/53) of the patients were prescribed 60 Gy in 27 fractions to rGTVnx, which did not significantly reduce the local control rate of the patients, but decreased the incidence of fatal complications caused by a higher total dose of reirradiation so that they may have longer survival [6]. Nonetheless, how to avoid severe radiation-related toxicities needs to be addressed. Thus, it is prudent to explore a novel salvage local treatment for locally advanced rNPC. Recently, the development of endoscopic surgery has made it possible to resect locally advanced disease without causing severe complications. Wong et al. reported 15 patients with rT_3-4_ rNPC receiving salvage ENPG and their 2-year OS rate was 66.7% [17]. Li et al. retrospectively analyzed 120 patients with locally advanced rNPC undergoing salvage ENPG, and their 3-year OS rate was 55.2% [16]. These encouraging results indicate that patients with locally advanced rNPC undergoing ENPG may achieve favorable survival, but it is unclear whether salvage ENPG is not inferior to IMRT. The recently published data of Li et al. [26] tried to answer this question by comparing salvage ENPG with IMRT and 3D-CRT in patients with locally advanced rNPC. However, the results should be interpreted cautiously due to potential confounding factors. Firstly, it is a single-center study and the numbers (192 vs. 51 vs. 11) of three groups of patients were in great disparity. Meanwhile, they did not perform any matching to ensure comparability between the three groups of patients. Additionally, the median follow-up was 19 months (1–118 months) in their study, which was insufficient to observe survival endpoints. Therefore, we conducted, to the best of our knowledge, the first multicenter, case-matched study to compare ENPG with IMRT in efficacy and safety in treating locally advanced rNPC. We found that the patients in the ENPG group had similar OS, CSS, LRFFS and PFS but a lower incidence of severe adverse events compared with those in the IMRT group.

To better screen candidates for ENPG, we divided the patients into three subgroups in terms of their invaded structures and difficulty of resection: unresectable tumors, potentially resectable tumors and easily resectable tumors. Unresected tumors, invading critical structures such as the functional brain area and posterior cervical vertebra, are usually too extensive to resect and repair, so resection can be life-threatening. Easily resectable tumors are either superficial or located in the midline, e.g., ethmoid sinus and axillary sinus, so resection can be safe and efficacious. Perhaps it is controversial with regard to potentially resectable tumors. These tumors are usually adjacent to or even involve important blood vessels and nerves or natural barriers, such as the ICA, CS, optic nerve and meninges, which require exquisite surgical skills and accessory instruments (stent or embolization) to prevent undesirable damage. Therefore, operating on this subgroup of patients will be greatly dependent on surgeons’ experience and skills and a qualified multidisciplinary team (skull base surgery, vascular intervention, neurosurgery, radiology, etc.) is needed. On the other hand, although entire resection is technically feasible, surgeons also take patients’ quality of life into consideration so that they will still retain these structures. After gross tumors are removed, the introduction of adjuvant treatment, e.g., chemotherapy or immunotherapy, may help to inhibit the proliferation of tumor cells or even eliminate residual tumors.

The patients in the ENPG group had even more favorable outcomes than those reported in previous studies, with a 3-year OS rate of 68.4% in our study; several factors may account for this finding. First, the advancement of medical instruments provided superior conditions for effective and safe endoscopic resection. For example, navigation can help surgeons recognize anatomical structures and avoid injury to critical vessels and nerves; low-temperature plasma technology can facilitate hemostasis and keep the operative field clear. ICA invasion is an adverse prognosticator and the 3-year OS rates of patients with or without ICA invasion were 15.7% and 65.1%, respectively [16]. The embolization of the ICA or the use of film-coated stents in our study made it possible to resect the tumor involving the ICA and reduce the rate of residual tumor and fatal ICA hemorrhage, which may be another contributor to favorable outcomes; this is also consolidated by the same study that the 3-year OS rate of patients with ICA invasion undergoing embolization and resection of the ICA has been improved from 15.7% to 100%. The third contributor to favorable prognosis was that reconstruction of the skull base was performed on a higher proportion (35/53) of patients, as this procedure can protect the wound, accelerate the healing of the operative area and reduce the risk of postoperative bleeding. The decrease in fatal perioperative complications may transform into a survival benefit.

The survival rate of the ENPG group was similar to that of the IMRT group; except for the improvement in surgical techniques and skill, we should not neglect the influence of selection bias and potential noncomparability of some characteristics. First, patients with unresectable tumors usually have a wide extent of tumor invasion and were excluded from the ENPG group, which may result in prolonged survival compared with the total population in this group. Second, although the rT category was balanced and the statistical results of resectability characteristics indicated that the distribution of involved structures was not significant different between the two groups, in the IMRT group, there was a higher number of petrous apex, ICA and cavernous sinus involvement, which may lead to survival outcomes favoring ENPG. Third, although we matched the baseline characteristics of the patients between the ENPG and IMRT groups with the PSM method, a disparity in N stage still existed between the two groups; e.g., the proportion of patients in N_0_ stage was higher in the ENPG group than in the IMRT group (70.8% vs. 62.2%), as lower N stage was a favorable prognosticator for rNPC. We also noticed that a higher proportion of patients in the ENPG group received subsequent treatment (56.6% vs. 41.5%) when they experienced tumor progression after salvage treatment, which we speculated may play a vital role in prolonging survival.

Although advances in medical instruments and endoscopic surgery techniques have greatly enhanced the tumor resection rate of rNPC, in this study, the positive rate of surgical margins and/or postoperative imaging of patients was 71.7%, which was similar to that reported by Chan et al. (71.4% in clivus bone invasion and 80.0% in sphenoid sinus lateral wall invasion) [27]. Some studies also report different results in that the negative surgical margin was shown to range from 50% to 70.8% [16, 17]. The high positive rate of surgical margins in our study may be due to the following reasons. First, it was difficult to distinguish tumor invading bones from radiation-induced osteitis, and the resected bones could not be examined by intraoperative frozen sections. Second, to avoid causing severe cerebrospinal fluid leakage, we performed palliative resection of lesions adjacent to the dura mater behind the clivus. Third, we performed palliative resection of CS lesions to avoid damage to important structures such as optic nerves. Fourth, we palliatively resected lesions involving cranial nerves and brain tissues to ensure the quality of life of patients after the operation. Finally, even if the surgical margin is negative, it is difficult to achieve a safe surgical margin of 0.5–1.0 cm outside the tumor [28].

Despite the high positive rate of surgical margins in our study, the patients in the ENPG group could still achieve long-term survival by using adjuvant treatment or re-salvage ENPG or IMRT. We noticed that 5 patients received ENPG twice or more times after tumor progression, which indicated that compared with salvage IMRT, ENPG could be repeatedly performed on patients as long as they were still suitable for surgery. Meanwhile, another advantage of ENPG over IMRT was that it could resect radioresistant tumors and acquire histological specimens to guide subsequent treatment. These aforementioned traits of ENPG avoided the accumulation of radiation-induced toxicities.

No grade 3 or worse nasal adhesion occurred in the ENPG group; it may be that the endoscopic transnasal pterygoid process approach was mostly used and that the middle and inferior turbinates had been removed due to the extensive lesions. In addition, the incidence of severe treatment-related complications in patients in the ENPG group was lower than in the IMRT group (37.7% vs. 67.9%, *P* = 0.02), indicating that ENPG may be a safe method for the treatment of locally advanced rNPC.

Our study has some limitations. Initially, due to the intrinsic nature of retrospective studies, the clinical data of the patients during or after treatment were difficult to record completely. In addition, doctors’ experience and skills could also affect the therapeutic quality of each procedure. Finally, although we adjusted for some prognostic factors between the ENPG and IMRT groups by the PSM method, potential noncomparability of some characteristics and selection bias between groups still existed, e.g., the exclusion of patients with unresectable tumors in the ENPG arm, resectability and the adjuvant therapy; this is expected to be solved by designing prospective, random controlled clinical studies in the future.

## Conclusions

Altogether, this study shows that salvage ENPG can obtain comparable oncological outcomes but less toxicities than IMRT for carefully screened patients with locally advanced rNPC. However, the potential noncomparability of some characteristics, e.g., resectability and selection bias between groups should not be neglected. Our study may provide some insights into the management of patients with locally advanced rNPC in which salvage ENPG may be a new local treatment option for this subgroup. Large, prospective and random controlled clinical trials should be performed to prove our findings.

### Supplementary Information

**Additional file 1**. Distribution of patients in each research center.

**Additional file 2**. Baseline demographics and clinical characteristics of the patients before matching.

## Data Availability

The data of our study can be accessed from corresponding author upon reasonable request.

## Abbreviations

rNPC: Recurrent nasopharyngeal carcinoma
IMRT: Intensity-modulated radiotherapy
ENPG: Endoscopic nasopharyngectomy
RT: Radiotherapy
3D-CRT: Three dimensional conformal radiotherapy
OAR: Organ at risk
OS: Overall survival
ICA: Internal carotid artery
CS: Cavernous sinus
RFI: Recurrence-free interval
PSM: Propensity scores matching
rGTV: Recurrent gross tumor volume
rCTV: Recurrent clinical target volume
LRFFS: Locoregional failure free survival
PFS: Progression free survival
CSS: Cancer-specific survival
IQR: Inter-quantile range
BOT: Balloon occlusion test
