# Supplementary material for: Endoscopic surgery versus intensity-modulated radiotherapy in locally advanced recurrent nasopharyngeal carcinoma: a multicenter, case-matched comparison
Source: J Otolaryngol Head Neck Surg. 2023 Nov 6;52:72. doi: 10.1186/s40463-023-00656-3 (PMC10629026; doi:10.1186/s40463-023-00656-3)
Supplement: Supplementary file 1 — Additional file 1. Distribution of patients in each research center. [file 40463_2023_656_MOESM1_ESM.docx]

**Supplementary Table 1.** Distribution of patients in each research center.

| **Cities** | **Centers** | **Investigators** | **Total patients** | **Matched patients** |
| --- | --- | --- | --- | --- |
| Guangzhou | Sun Yat-sen University Cancer Center | Fei Han | 102 | 53 |
| Guangzhou | Guangdong Provincial People's Hospital | Qianhui Qiu | 12 | 9 |
| Guangzhou | Zhujiang Hospital, Southern Medical University | Liangcai wan | 53 | 38 |
| Wuzhou | Wuzhou Red Cross Hospital | Bin He | 8 | 6 |
| Total |  |  | 175 | 106 |
