# Supplementary material for: Endoscopic surgery versus intensity-modulated radiotherapy in locally advanced recurrent nasopharyngeal carcinoma: a multicenter, case-matched comparison
Source: J Otolaryngol Head Neck Surg. 2023 Nov 6;52:72. doi: 10.1186/s40463-023-00656-3 (PMC10629026; doi:10.1186/s40463-023-00656-3)
Supplement: Supplementary file 2 — Additional file 2. Baseline demographics and clinical characteristics of the patients before matching. [file 40463_2023_656_MOESM2_ESM.docx]

| **Characteristics** | **Overall** | **ENPG** | **IMRT** | **P-value** |
| --- | --- | --- | --- | --- |
|  | **NO. (%)** | **NO. (%)** | **NO. (%)** |  |
| No. of Patients | 176 | 74 | 102 |  |
| Sex |  |  |  | 0.480 |
| Male | 140 (79.5) | 57 (77.0) | 83 (81.4) |  |
| Female | 36 (20.5) | 17 (23.0) | 19 (18.6) |  |
| Median age at recurrence, years (IQR) | 48 (40-55) | 50 (40-57) | 47 (41-52) | 0.099 |
| Median recurrent interval, months (IQR) | 39 (20-84) | 39 (15-88) | 38 (20-68) | 0.587 |
| Histology |  |  |  | <0.001 |
| WHO type II+III | 143 (81.2) | 48 (64.9) | 95 (93.1) |  |
| WHO type I+other | 33 (18.8) | 26 (35.1) | 7 (6.9) |  |
| Recurrent T classification |  |  |  | 0.305 |
| rT3 | 103 (58.5) | 40 (54.1) | 63 (61.8) |  |
| rT4 | 73 (41.5) | 34 (45.9) | 39 (38.2) |  |
| Recurrent N classification |  |  |  | 0.030 |
| rN0 | 119 (67.5) | 59 (79.6) | 60 (58.9) |  |
| rN1 | 39 (22.2) | 3 (4.1) | 36 (35.3) |  |
| rN2 | 14 (8.0) | 11 (14.9) | 3 (2.9) |  |
| rN3 | 4 (2.3) | 1 (1.4) | 3 (2.9) |  |
| Systemic treatment |  |  |  | 0.002 |
| Yes | 107 (60.8) | 55 (74.3) | 52 (51.0) |  |
| No | 69 (39.2) | 19 (25.7) | 50 (49.0) |  |

**Supplementary Table 2**. Baseline demographics and clinical characteristics of the patients before matching.

IQR, Inter quartile range; ENPG, endoscopic nasopharyngectomy; IMRT, intensity-modulated radiotherapy.
